# Exercise training improves intramuscular triglyceride lipolysis sensitivity in high-fat diet induced obese mice

**DOI:** 10.1186/s12944-018-0730-8

**Published:** 2018-04-16

**Authors:** Kangeun Ko, Jinhee Woo, Ju Yong Bae, Hee Tae Roh, Yul Hyo Lee, Ki Ok Shin

**Affiliations:** Laboratory of Exercise Biochemistry, Department of Physical Education, College of Arts and Physical Education, Dong-A University, 37 Nakdong-daero 550 beon-gil, Hadan-dong, Saha-gu, Busan, 49315 Republic of Korea

**Keywords:** High-fat diet, Exercise training, Intramuscular triglyceride lipolysis, Obesity

## Abstract

**Background:**

The purpose of this study was to determine whether regular exercise training enhances intramuscular triglyceride (IMTG) lipolysis sensitivity during consumption of a continued high-fat diet by exploring changes in biochemical factors activated by IMTG lipolysis.

**Methods:**

Male C57BL/6 mice aged 4 weeks were randomly divided into a high-fat diet group (HF) to induce obesity for 6 weeks and a control (CO) group. Thereafter, the HF group was divided into a high-fat diet group (HF) and high-fat diet + training group (HFT). The HFT group was trained on an animal treadmill 40 min/day, 5 days/week for 8 weeks. PKA, Plin5, p-Plin5, CGI-58, ATGL, and HSL were analyzed to investigate IMTG sensitivity by western blotting.

**Results:**

PKA, CGI-58, and HSL protein levels in the HF group were significantly lower than those in the CO group (*p* < 0.05). However, PKA, CGI-58, and HSL protein levels in the HFT group were significantly higher than those in the HF group, and ATGL and p-Plin5 protein levels as well as the p-Plin5/Plin5 ratio in the HFT group were significantly higher than those in the HF group (*p* < 0.05). In addition, the HF group showed a significantly higher IMTG volume than the CO and HFT groups (*p* < 0.05).

**Conclusions:**

These results suggest that in an obese mouse model, 8 weeks of treadmill exercise contributes to decreased IMTG volume by activating lipolysis factors, such as PKA, PLIN5, CGI-58, and lipases. Therefore, regular exercise training may play an important role in obesity treatment by increasing IMTG lipolysis sensitivity.

## Background

Excessive accumulation of adipose tissue caused by energy imbalance limits the storage capacity of lipids in adipose tissue and increases the accumulation of fat in other tissues, including the liver, muscle, and heart [1]. This ectopic fat can increase the risk of insulin resistance, type 2 diabetes, and cardiovascular diseases [2]. Additionally, triglyceride (TG) levels within the skeletal muscle, or intramuscular triglyceride (IMTG), which exists in healthy muscles in small quantities, is increased in obese individuals [3].

TGs are stored in lipid droplets (LD) with cholesteryl esters enclosed by a phospholipid monolayer. The LD surface contains many proteins [4], and these coat proteins are known to play an essential role in the metabolic regulation of IMTG and reduce lipid-induced insulin resistance and lipotoxicity [5]. Particularly, the best-characterized family of LD coat proteins is the perilipin (Plin) protein family, which is composed of five members (Plin1–5) [6], each of which has a unique tissue distribution. Plin5, also known as LSDP-5, MLDP, PAT-1, and OXPAT, is mostly expressed in tissues with high oxidative capacity such as the skeletal muscle [7, 8]. Currently, the function of Plin5 in LD breakdown is not fully understood. Plin5 in the skeletal muscle is expected to have a similar role to Plin1, which is well-known to be expressed only in adipose tissues [9]. Previous studies showed that Plin5 binds adipose triglyceride lipase (ATGL), which is the rate-limiting enzyme in TG hydrolysis, and comparative gene identification-58 (CGI-58, also known as ABHD5), a powerful activator of ATGL; although the lipolysis activity of ATGL is reduced, Plin5 expression recruits ATGL to the LD surface [10]. However, Plin5 phosphorylation promotes the release of CGI-58 to bind ATGL from the Plin scaffold under cAMP-dependent protein kinase (PKA) activation, and the interaction of CGI-58 with ATGL activates lipolysis. Moreover, hormone-sensitive lipase (HSL), which is the rate-limiting enzyme in diglyceride to monoglyceride hydrolysis, interacts with Plin5 [11].

Physical inactivity caused by a sedentary lifestyle can reduce energy consumption and increase vulnerability to various illnesses, including obesity. IMTG use as an energy source is promoted when energy needs are increased through exercise [12]. The high rate of IMTG breakdown during exercise improves IMTG turnover and prevents the accumulation of lipotoxic molecules [13]. IMTG breakdown also helps maintain insulin sensitivity. Therefore, it is important to understand the mechanisms regulating IMTG lipolysis.

However, few studies have examined how the metabolism pathway related to LD coat proteins changes with IMTG lipolysis caused by exercise. Particularly, the relationship between exercise and Plin5 expressed in the skeletal muscle has not been thoroughly evaluated.

Therefore, the aim of this study was to determine whether regular exercise training enhances IMTG lipolysis sensitivity during consumption of a continued high-fat diet, which can cause energy metabolic confusion, by exploring changes in biochemical factors activated by IMTG lipolysis.

## Methods

### Animals and diets

Twenty-four C57BL/6 male mice (4 weeks old) were obtained from Samtako Bio Korea (Gyeonggi-do, Korea) and raised under controlled conditions including temperature (22–24 °C), humidity (55–60%), and light (12 h light/dark cycle) at the Dong-A University College of Medicine Animal Laboratory. After 1 week of adaptation maintenance and free access to food and water, the mice were randomly divided into two groups: control group (CO, *n* = 8) and high-fat diet group (HF, *n* = 16). To induce obesity, CO mice were fed a normal diet (9.41% carbohydrate, 6.52% fat, 24.34% protein, Research Diets, Inc., New Brunswick, NJ, USA), while HF mice were fed a high-fat diet (35% carbohydrate, 45% fat, 20% protein, Samtako, Inc.) for 6 weeks. After inducing obesity, the HF group was divided into two groups; HF (*n* = 8) and HF + training (HFT, *n* = 8), and the HFT was subjected to treadmill training for 8 weeks. All animal procedures were approved by the Dong-A University Medical School Institutional Animal Care and Use Committee. This experiment was conducted according to committee guidelines.

### Exercise training

HFT mice were trained on an animal treadmill for 40 min/day, 5 days/week for 8 weeks using a modified exercise protocol described previously [14]. The exercise load comprised of 5 m/min for 5 min (warm-up and cool-down) and 10 m/min for 30 min (exercise training) at 0 grade inclination for the first 4 weeks of mild intensity session. For weeks 5–8, the exercise load conducted at 5 m/min for 5 min (warm-up and cool-down) and 14 m/min for 30 min (exercise training) at the same incline (moderate intensity). This exercise intensity was set according to the maximum oxygen consumption of C57BL/6 mice and muscle TG energy expenditure ratio [15, 16].

### Samples of blood and tissue

Animals were sacrificed at 48 h after the end of training to rule out the temporary effects of exercise training. All mice were anesthetized by using ethyl ether. Next, blood was collected from the abdominal aorta, and the soleus muscle was removed. Serum was obtained by centrifugation at 3000 rpm for 10 min. Blood and soleus muscle were immediately stored at − 80 °C.

### Analysis content

#### Lipid profiles

Serum profiles were analyzed by using total cholesterol (TC), TG (Asan Pharmaceutical, Seoul, Korea), and high-density lipoprotein cholesterol (HDL-c) kits (Shinyang Diagnostics, Siheung-si, Korea). Low-density lipoprotein cholesterol (LDL-c) was calculated using a previously described formula [17].

Muscle triglyceride content was analyzed by ethanolic potassium hydroxide (KOH) saponification using the soleus muscles as described previously [18, 19]. In summary, muscle samples were homogenized in ethanolic KOH (2 parts ethanol: 1 part 30% KOH) and incubated overnight at 55 °C. These samples were mixed with 50% ethanol (H_2_O: ethanol = 1: 1) and centrifuged for 5 min at 13,000 rpm. The supernatant was mixed with 1 M MgCl_2_ and incubated for 10 min on ice, followed by centrifugation for 10 min at 4 °C, 13,000 rpm. The supernatant was used to measure TG contents using ASAN set Triglyceride-S Reagent (AM157S-K, Asan Pharmaceutical).

#### Western blotting

Soleus muscle samples were extracted using radioimmunoprecipitation assay buffer (Tris-HCl, pH 8.0, 150 mM sodium chloride, 1.0% Igepal CA-630 (NP-40), 0.5% sodium deoxycholate, 0.1% sodium dodecyl sulfate, protease inhibitor cocktail, and phosphatase inhibitor cocktail). The proteins were resolved by 10 or 12% SDS-polyacrylamide gel electrophoresis and transferred to polyvinylidene difluoride membranes. The membranes were blocked overnight with 5% skim milk in PBST (8 g NaCl, 0.2 g KCl, 1.44 g Na_2_HPO_4_, 0.24 g KH_2_PO_4_, pH 7.4, 1% Tween-20) and then incubated for more than 2 h with primary antibody (PKA; sc-98,951, Santa Cruz Biotechnology, Dallas, TX, USA, Plin5; sc-240,627, Santa Cruz Biotechnology, p-PKA substrate; 9621 s, Cell Signaling Technology, Danvers, MA, USA, CGI-58; sc-100,468, Santa Cruz Biotechnology, ATGL; sc-67,355, Santa Cruz Biotechnology, HSL; sc-25,843, Santa Cruz Biotechnology). Membranes were developed using horseradish peroxidase-conjugated anti-goat, mouse, or rabbit IgG, followed by incubation with ECL solution (Amersham Pharmacia Biotech, Piscataway, NJ, USA), followed by detection using a Fuji LAS-4000 Imaging station (ImageQuantTMLAS-4000, GE Healthcare, Little Chalfont, UK).

### Statistical analysis

The data were analyzed using SPSS windows version 22.0 software (SPSS, Inc., Chicago, IL, USA), and all measurements were presented as the means ± standard error (SE).

The independent sample t-test was used to detect significant differences in body weights during the obesity induction period. In addition, statistical analysis of the body weight, lipid profiles, and protein levels after treadmill training was conducted by one-way analysis of variance. The statistical significance level was identified through the Tukey’s post hoc test, and all differences were considered significant when the p-value was less than 0.05.

## Results

### Changes in body weight

**Fig. 1:**
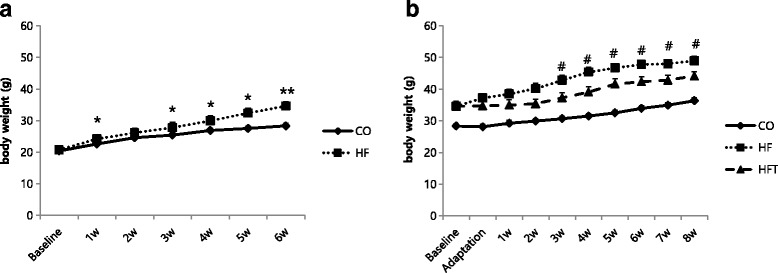
Changes in body weight for each experimental treatment period. Changes in body weight during 6 weeks of obesity induction period (**a**) and during 8 weeks of training period (**b**). CO; Control group, HF; High-fat diet group, HFT; High-fat diet + Training group. Values are presented the means ± SE. ^*^p < 0.05, ^**^p < 0.01; Significant difference from the CO group, ^#^ *p* < 0.05; Significant difference from the HFT group

The changes in body weight during the obesity induction period are presented in Fig. 1a. A difference in body weight between groups was observed at 1 week after starting the high-fat diet (1.59 g, *p* < 0.05), and this difference was significantly increased at 6 weeks after high-fat diet feeding (6.26 g, *p* < 0.01).

Changes in body weight during the 8 weeks of exercise training are presented in Fig. 1b. The body weight of the HFT group was significantly lower than that in the HF group after 3 weeks of training (5.56 g, *p* < 0.05), and this tendency was maintained until the end of the training period (4.86 g, *p* < 0.05).

### Changes in lipid profiles

Table 1 presents the changes in serum lipid profiles. Serum TG, TC, and LDL-c in the HF group were significantly higher than those in the CO and HFT groups (*p* < 0.05). Serum TG, TC, and HDL-c in the HFT group was significantly higher than that in the CO group (*p* < 0.05).

**Table 1:** Lipid profiles of the subjects

Variable	CO (*n* = 8)	HF (*n* = 8)	HFT (*n* = 8)
TG (mg/dL)	86.37 ± 2.23	121.49 ± 6.98^*^	104.09 ± 3.73^*,#^
TC (mg/dL)	98.78 ± 3.08	193.74 ± 12.93^*^	158.10 ± 8.80^*,#^
HDL-c (mg/dL)	42.03 ± 4.16	55.17 ± 4.51	62.44 ± 7.70^*^
LDL-c (mg/dL)	39.47 ± 4.84	114.27 ± 11.78^*^	74.84 ± 11.68^#^

Values are presented the means ± SE. *TG* triglyceride, *TC* Total cholesterol, *HDL-c* high-density lipoprotein cholesterol, *LDL-c* low-density lipoprotein cholesterol, *CO* control group, *HF* high-fat diet group, *HFT* high-fat diet + training group

^*^Significant difference from the CO group (*p* < 0.05)

^#^Significant difference from the HF group (*p* < 0.05)

### Protein levels

**Fig. 2:**
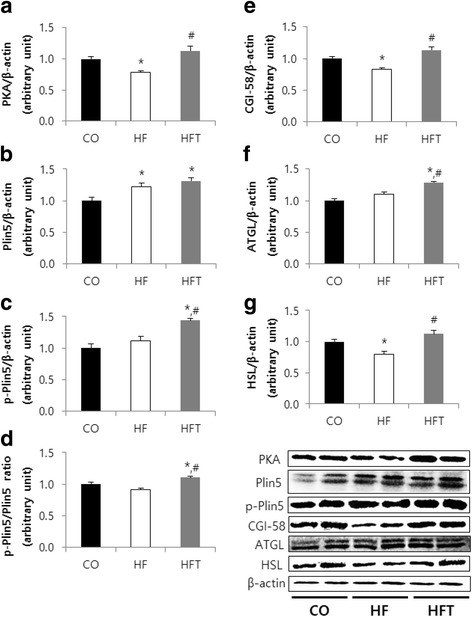
Effects of exercise training on lipolysis pathway factors in soleus muscle. Comparison of protein levels of PKA (**a**), Plin5 (**b**), p-Plin5 (**c**), p-Plin5/Plin5 ratio (**d**), CGI-58 (**e**), ATGL (**f**), and HSL (**g**) after 8 weeks of training. CO; Control group, HF; High-fat diet group, HFT; High-fat diet + Training group. Values are presented the means ± SE. ^*^p < 0.05; Significant difference from the CO group, ^#^ *p* < 0.05; Significant difference from the HF group

We explored IMTG lipolysis pathway-related factors in the soleus muscle to determine whether lipolysis sensitivity is improved by exercise training in obese mice. The PKA protein level in the HF group was significantly lower than that in the CO and HFT groups (*p* < 0.05) (Fig. 2a).

The Plin5 protein level in the HF and HFT groups was significantly higher than that in the CO group (*p* < 0.05) (Fig. 2b). The p-Plin5 protein level and p-Plin5/Plin5 ratio in the HFT group was significantly higher than that in the CO and HF groups (*p* < 0.05) (Fig. 2c, d).

The CGI-58 and HSL protein levels in the HF group was significantly lower than that in the CO and HFT groups (*p* < 0.05) (Fig. 2e, g). The ATGL protein level in the HFT group was significantly higher than that in the CO and HF groups (*p* < 0.05) (Fig. 2f).

**Fig. 3:**
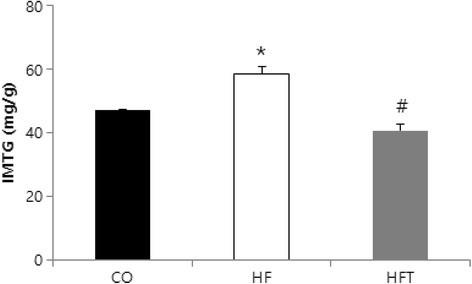
Effects of exercise training on IMTG volume in soleus muscle. CO; Control group, HF; High-fat diet group, HFT; High-fat diet + Training group. Values are presented the means ± SE. *p < 0.05; Significant difference from the CO group, # *p* < 0.05; Significant difference from the HF group

The IMTG content of the HF group was significantly higher than that of the CO and HFT groups (*p* < 0.05) (Fig. 3).

## Discussion

Plin is phosphorylated by activation of PKA following accumulation of intracellular cAMP under β-adrenergic stimulation during fasting or exercise. The activation of ATGL is promoted by its interaction with CGI-58 released from the Plin scaffold [10, 20]. Plin phosphorylation is essential for shifting HSL from the cytosol to the LD surface, where it is activated [20, 21]. The results of this study showed that the PKA protein levels in the HF group were significantly lower than those in the CO and HFT group. This may be because sympathetic nerve stimulation by exercise contributed to the metabolic pathway of TG decomposition by increasing PKA, which initiates TG decomposition.

This study showed that the Plin5 protein levels in the HF and HFT groups were significantly higher than that in the CO group. This result corresponds to those of similar studies, which showed that Plin5 protein levels in low-fat or normal diet groups were significantly higher than in HF and HF + active groups [22–24]. The levels of the lipid droplet coat protein Plin5 may be related to the increase in lipid droplets, as fat mass is increased by consumption of a high-fat diet. Phosphorylated Plin5 levels in the HFT group were significantly higher than those in the CO and HF groups. This may be induced by increases in PKA protein levels and contribute to the activation of lipases. Macpherson et al. [25] investigated the effects of epinephrine and intermittent tetanic stimulation on serine phosphorylated Plin5 in isolated skeletal muscle. However, the effects of aerobic exercise on in vivo levels of Plin5 phosphorylation have not been investigated. This study examined changes in phosphorylated Plin5 using a phospho-PKA substrate antibody, as Plin5 is a PKA substrate. Therefore, further in vivo studies are necessary, as well as additional follow-up research to confirm the specific site of regular exercise-induced phosphorylation on Plin5.

As described above, phosphorylated Plin5 facilitates TG breakdown to active CGI-58 and ATGL [10]. TG breakdown is promoted by lipases such as ATGL and HSL, which are essential for effective lipolysis in the skeletal muscle [26]. CGI-58, as a coactivator of ATGL, plays an important role in regulating the activity of ATGL [27]. Macpherson et al. [25] reported that the interaction between ATGL and CGI-58 was increased following contraction. CGI-58 protein levels in the HF group were significantly lower than those in the CO group, although the ATGL protein level in the HF group was not changed. CGI-58 and ATGL protein levels were significantly higher in the exercise training group than in the group that only consumed a high-fat diet. This suggests that a high-fat diet can reduce lipolytic activity by lowering the levels of CGI-58, an activator of ATGL, while regular exercise training can facilitate lipolysis by increasing the levels of factors that activate the first step of IMTG decomposition.

The genetic inactivity of ATGL increases TG levels in many tissues [28]. Badin et al. [29] reported that muscle ATGL protein was increased in obesity while muscle HSL protein was reduced, and that HSL inhibition induces insulin resistance and diacylglycerol (DAG) accumulation. ATGL overexpression mediates adenovirus-induced ceramide and DAG accumulation and disrupts insulin signaling [29]. The balance of ATGL and HSL in the skeletal muscle plays an important role in insulin signaling and activity, as these defects were reinstated by HSL overexpression [29]. HSL protein levels in the HF group were significantly lower than those in the CO group. ATGL and HSL protein levels in the HFT group were also significantly higher than those in the HF group. In agreement with the results of Badin et al. [29], this suggests that an imbalance in ATGL and HSL caused by decreased HSL can lead to metabolic disorders through the accumulation of lipotoxic molecules such as DAG and ceramide. However, increased PKA protein levels caused by exercise training may activate Plin5, which may result in increased levels of lipases such as ATGL and HSL. This may reduce the negative influence on metabolic disorders and contribute to improving IMTG lipolysis sensitivity by decreasing the accumulation of lipotoxic molecules.

TG in the skeletal muscle is used as an energy source and may be increased by endurance training exercise [30, 31]. However, obesity and type 2 diabetes are also related to increased IMTG levels [3], and IMTG can lead to insulin resistance caused by high levels of lipotoxic molecules [32]. IMTG content in the HF group was significantly higher than that in the CO and HFT groups. This result corresponds with the studies mentioned above. Interestingly, blood TG levels were shown to be significantly higher in the HFT group than in the CO group, while there was no significant difference in IMTG levels between the two groups. This suggests that the extent of change in TG levels may differ between the blood and muscle. High blood TG levels are thought to be caused by TG secretion from excessive subcutaneous adipose tissue, or by TG movement into the blood for transport to tissues that require TG. Taking into account that IMTG can be used as an energy source during exercise [33], the lack of difference in IMTG levels between the two groups may be because of its contribution as an energy source in the HFT group and was induced by regular exercise.

## Conclusion

In conclusion, the results of this study suggest that in an obese model, 8 weeks of treadmill exercise may improve blood lipids, and contribute to decreasing IMTG by activating lipolysis factors. Therefore, regular exercise training may play an important role in obesity treatment by increasing IMTG lipolysis sensitivity.

## Abbreviations

ATGL: Adipose triglyceride lipase
CGI-58: Comparative gene identification-58
DG: Diglyceride
HDL-c: High density lipoprotein cholesterol
HF: High-fat diet group
HFT: High-fat diet plus exercise training group
HSL: Hormone sensitive lipase
IMTG: Intramuscular triglyceride
LD: Lipid droplets
LDL-c: Low density lipoprotein cholesterol
MG: Monoglyceride
ND: Normal diet group
PKA: cAMP-dependent protein kinase A
Plin: Perilipin
p-Plin: Phosphorylation perilipin
TC: Total cholesterol
TG: Triglyceride
